# Over-expression of an electron transport protein OmcS provides sufficient NADH for d-lactate production in cyanobacterium

**DOI:** 10.1186/s13068-021-01956-4

**Published:** 2021-04-29

**Authors:** Hengkai Meng, Wei Zhang, Huawei Zhu, Fan Yang, Yanping Zhang, Jie Zhou, Yin Li

**Affiliations:** 1 Department of Cellular Biology, University of Science and Technology of China, Hefei, China; 2 CAS Key Laboratory of Microbial Physiological and Metabolic Engineering, State Key Laboratory of Microbial Resources, Institute of Microbiology, Chinese Academy of Sciences, No. 1 West Beichen Road, Chaoyang District, Beijing, 100101 China; 3 University of Chinese Academy of Sciences, Beijing, China; 4 State Key Laboratory of Transducer Technology, Institute of Microbiology, Chinese Academy of Sciences, Beijing, 100101 China

**Keywords:** Cyanobacteria, Electron transport protein, Photosynthetic electron, Electron transfer, NADH availability, Lactate production

## Abstract

**Background:**

An efficient supply of reducing equivalent is essential for chemicals production by engineered microbes. In phototrophic microbes, the NADPH generated from photosynthesis is the dominant form of reducing equivalent. However, most dehydrogenases prefer to utilize NADH as a cofactor. Thus, sufficient NADH supply is crucial to produce dehydrogenase-derived chemicals in cyanobacteria. Photosynthetic electron is the sole energy source and excess electrons are wasted in the light reactions of photosynthesis.

**Results:**

Here we propose a novel strategy to direct the electrons to generate more ATP from light reactions to provide sufficient NADH for lactate production. To this end, we introduced an electron transport protein-encoding gene *omcS* into cyanobacterium *Synechococcus elongatus* UTEX 2973 and demonstrated that the introduced OmcS directs excess electrons from plastoquinone (PQ) to photosystem I (PSI) to stimulate cyclic electron transfer (CET). As a result, an approximately 30% increased intracellular ATP, 60% increased intracellular NADH concentrations and up to 60% increased biomass production with fourfold increased d-lactate production were achieved. Comparative transcriptome analysis showed upregulation of proteins involved in linear electron transfer (LET), CET, and downregulation of proteins involved in respiratory electron transfer (RET), giving hints to understand the increased levels of ATP and NADH.

**Conclusions:**

This strategy provides a novel orthologous way to improve photosynthesis via enhancing CET and supply sufficient NADH for the photosynthetic production of chemicals.

**Supplementary Information:**

The online version contains supplementary material available at 10.1186/s13068-021-01956-4.

## Background

Cyanobacteria are photoautotrophic prokaryotes capable of converting ambient CO_2_ into organic compounds using sunlight as the energy source. Engineering cyanobacteria for chemical production draws increasing attention in recent years with the hope to use CO_2_ as an alternative feedstock [1–3]. To date, engineered cyanobacteria have been reported to produce more than 30 chemicals from CO_2_ [4, 5]. However, the titer and the productivity of the target chemical are usually two orders of magnitude lower than that of the same chemical produced by heterotrophic microorganisms using sugar as a carbon source.

Cofactor availability, in particular the availability of reducing equivalent (in the form of NADH/NADPH) and ATP, is crucial to microbial production of chemicals. In most heterotrophic microorganisms, NADH and ATP are generated from the catabolism of glucose, and NADH is the dominant form of the reducing equivalent [6]. While in cyanobacteria and other phototrophic microorganisms, NADPH and ATP are generated from photosynthetic electrons derived from sunlight in the light reactions of photosynthesis, then the NADPH and ATP are used for CO_2_ fixation to synthesize organic carbon [7]. Lastly, NADH and ATP will be generated from the catabolism of organic carbon and ATP will also be generated from NADH via RET. As the molecules of NADPH generated from photosynthesis are much more than the NADH generated from catabolism, NADPH is the dominant form of reducing equivalent in cyanobacteria, and the NADH pool in cyanobacteria is lower than its NADPH pool under photoautotrophic conditions [8–11]. Consequently, NADH-dependent enzymes are less active than NADPH-dependent ones in cyanobacteria due to a shortage of NADH [12–14].

Dehydrogenases are involved in the biosynthesis of many chemicals [14, 15]. Most dehydrogenases in nature prefer to utilize NADH as a cofactor, indicating these NADH-dependent dehydrogenases might not work efficiently in cyanobacteria [12–14]. Several approaches have been developed to tackle this problem [14]. This includes finding and applying NADPH-dependent dehydrogenases [12, 13], using NADH-dependent dehydrogenases co-expressed with soluble transhydrogenase to convert NADPH into NADH [16, 17], or changing the cofactor preference of the dehydrogenases from NADPH to NADH through site-directed mutagenesis [15, 18]. All these approaches helped to increase the production of target chemicals in NADPH-rich cyanobacteria cells. Since NADPH is the main reducing equivalent used in cyanobacteria to fix CO_2_ through photosynthesis, over-consuming the NADPH might negatively affect cell growth and carbon fixation, and consequently affect the production of chemicals [9, 17].

In cyanobacteria, solar energy is firstly converted into photosynthetic electrons, which are subsequently converted into chemical energy ATP and NADPH via photosynthetic electron transfer. Linear electron transfer (LET) from photosystem II (PSII) to photosystem I (PSI) generates ATP and NADPH at a fixed ratio [19, 20]. Besides the LET, as auxiliary, cyclic electron transfer (CET) cycles electrons from the PSI acceptor side back to plastoquinone (PQ), cytochrome *b6f* (cyt*b6f*) complex and plastocyanin (PC), generating ATP without net NADPH production to meet metabolic demand [21]. Cyanobacteria also have RET, where the NADH generated from the catabolism of organic carbon donates electrons to generate ATP for sustaining cell growth or cellular activities [22]. On the other hand, up to 80% of the absorbed photons are dissipated to avoid photo-damage, when photosynthetic energy is much higher than that can be handled by photosynthetic electron transfer under high illumination conditions [23–25]. We, therefore, ask a question: if we could redirect the excess photoelectrons to generate more ATP, can we save the NADH originally used for ATP generation through RET, and direct the NADH to chemical production?

**Fig. 1:**
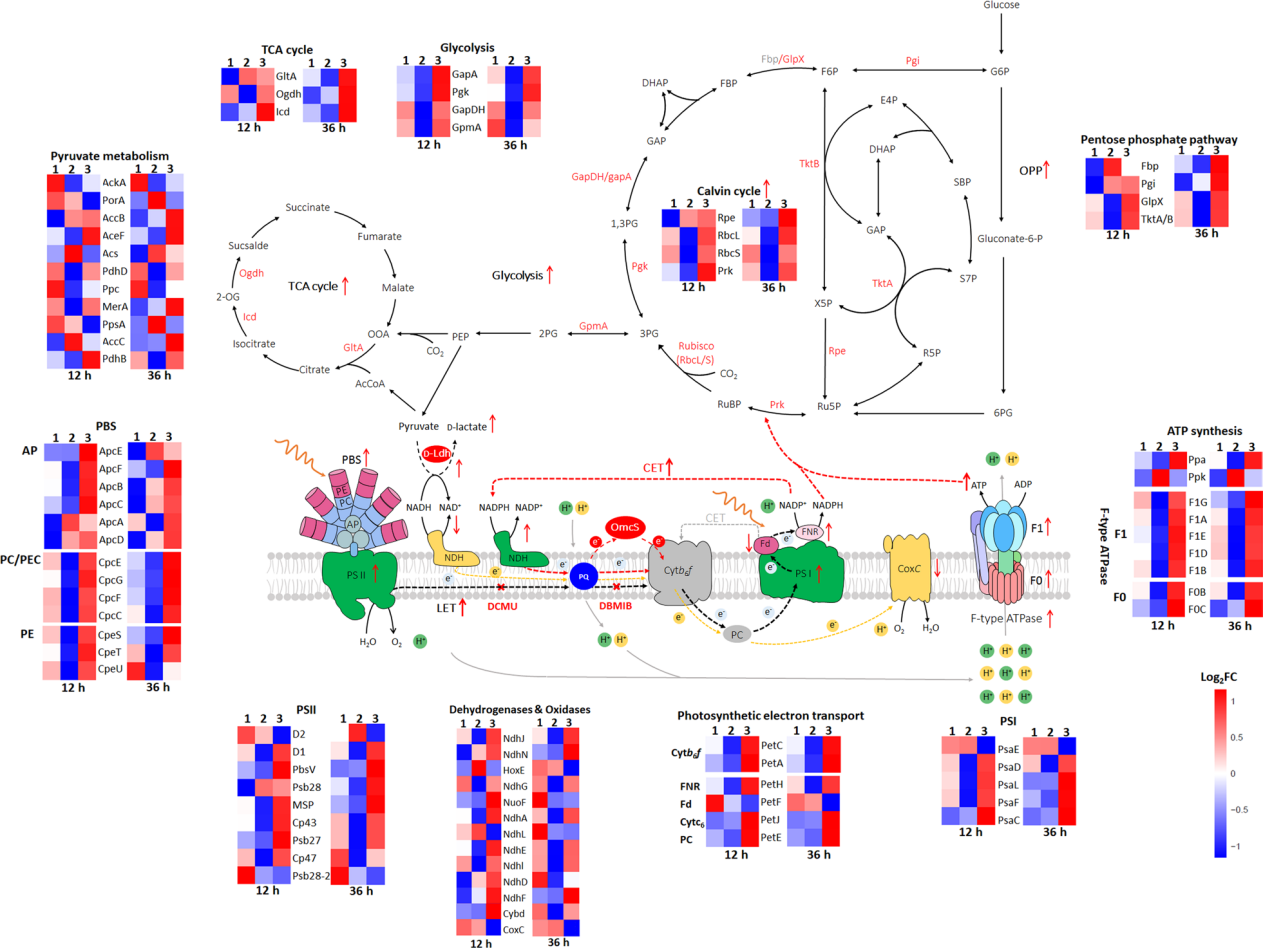
Effects of OmcS on the expression of central metabolism genes in Syn2973. Cells grew for 12 and 36 h, respectively. The Log_2_Ratio is shown for each gene on heat maps. 1, Syn2973; 2, Syn2973-Ldh; 3, Syn2973-LdhOmcS. The upregulated genes of each pathway are red. Black arrows represent LET, which generates ATP and NADPH coupled. Red arrows represent NDH-dependent CET and light grey arrows represent PGR5-dependent CET. Yellow arrows represent RET. To make it convenient for understanding, we use the protein name instead of the gene name

As the fast-growing cyanobacterium, *Synechococcus elongatus* UTEX 2973 (thereafter Syn2973) is a close relative of *Synechococcus elongatus* 7942 (Syn7942) with 99.8% identical genome sequences [26]. To test the hypothesis, an electron transport protein c-type outer membrane cytochromes (OmcS, from *Geobacter* sp.), which can be functionally expressed in Syn7942 as a soluble protein with a high ability to transport electrons [27], was introduced into strain Syn2973. We used d-lactate production by cyanobacteria as a model to test if such an approach can create more NADH which is required for d-lactate production (Fig. 1). A series of physiological, biochemical, electrochemical analyses and comparative transcriptome analyses demonstrated that the approach can increase the availability of NADH for lactate production and significantly increased the photosynthetic activity, indicating channeling photosynthetic electrons for generating more ATP is an efficient strategy for NADH-dependent dehydrogenases to function well in photosynthetic organisms.

## Results

### Experiment design and introducing OmcS

As already illustrated, compared with the NADPH directly generated from light energy, the NADH generated from the catabolic metabolism of organic carbon is the minor form of reducing equivalent in cyanobacteria. Generally, the NADH will be consumed by RET for ATP generation, leaving little NADH available for chemical production using NADH-dependent dehydrogenases [28, 29]. Therefore, increasing ATP generation from photosynthetic electrons in light reactions of photosynthesis might decrease ATP generation from RET and save NADH that otherwise would be consumed by RET. In the light reactions of photosynthesis, photosystem II (PSII) utilizes the absorbed photons to split H_2_O into O_2_, H^+^ and releases photosynthetic electrons to generate ATP and NADPH via photosynthetic electron transfer. In fact, up to 80% of the absorbed photons are dissipated from photosystems [23–25, 30]. Therefore, if the excess photosynthetic electrons can be used to generate more ATP, we hypothesize it might help to reduce the amount of NADH that is consumed in the RET (Fig. 1).

**Fig. 2:**
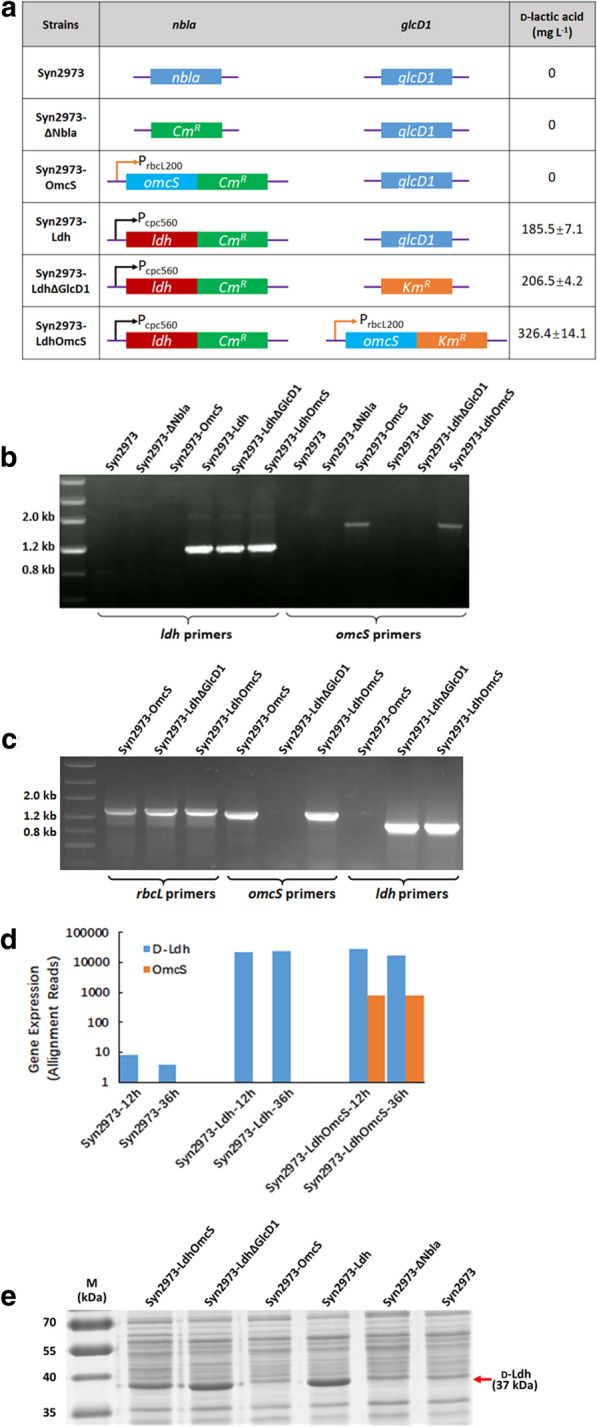
Construction of OmcS introduced and d-lactate-producing strains. **a** Genetic modifications and d-lactate production in all strains. **b** Genome PCR confirmed the integration of *omcS* and *Idh* genes into the genome of all mutants. **c** RT-PCR confirmation of expression of *omcS* and *ldh* in corresponding strains. Rubisco large subunit *rbcL* was used as positive control. **d** Comparable analysis of *omcS* and *ldh* gene expression in corresponding strains at 12 and 36 h during rapid d-lactate production period by transcriptome. **e** Ldh expression in corresponding strains by 12% SDS-PAGE analysis. Expression level of Ldh under control of strong promoter P_cpc560_ was approximately 10% of total soluble protein, whereas OmcS (47 kDa) expressed under control of P_rbcL200_ was almost undetectable on the gel

In thylakoid membrane, LET from PSII to PSI generates ATP and NADPH coupled from light energy for CO_2_ fixation, while CET around PSI generating ATP without net NADPH formation plays an important roles in balancing LET and CET and regulating the ratio of ATP and NADPH to acclimate to various environmental stresses, such as high light intensity and low organic carbon [20, 31–33]. Regulating LET and CET is crucial for efficient photosynthesis and enhancing CET can drive LET as well as photosynthesis [20, 31, 32]. As the most abundant mobile small electron carrier, PQ is shared by LET, CET, and RET in cyanobacteria [22, 34–36] (Fig. 1). Increasing the capability of accepting electrons of PQ or more oxidized PQ can attract more electrons from PSI and enhance CET [20, 21]. Thus in this work, we proposed that redirecting excess electrons from PQ might enhance CET and subsequently regulates the ratio of ATP and NADPH to meet the metabolic demand. Therefore, to generate more ATP from light reactions of photosynthesis, we attempted to select an electron carrier that can channel excess photosynthetic electrons from PQ to stimulate CET as well as to drive LET (Fig. 1). A redox protein called c-type outer membrane cytochromes OmcS from *Geobacter* sp. was expressed in Syn7942 [27] and the extracellularly electron transfer ability of the cells was increased. In this work, we hypothesized that OmcS may regulate the balance of LET and CET by transferring electrons from PQ. The balance of LET and CET needed to be finely regulated, and strong expression of OmcS might exert unfavorable perturbations to intracellular electron transfer. We, therefore, chose a weak promoter P_rbcL200_ for OmcS expression and inserted the electron carrier coding gene *omcS* into the *nbla* locus of the genome of cyanobacterium Syn2973 [26] to test if more ATP can be generated (Fig. 1). We designated the resulting strain as Syn2973-OmcS and used Syn2973*-*ΔNbla strain as its control, in which we inserted a chloromycetin expression cassette into the *nbla* locus of wild-type strain Syn2973 (Fig. 2a). PCR and sequencing verified the integration of *omcS* gene and full chromosome segregation (Fig. 2b). RT-PCR showed that the introduced *omcS* in strain Syn2973-OmcS is transcribed (Fig. 2c) and the expression level of OmcS response to different growth periods was analyzed via transcriptome (Fig. 2d).

### OmcS can direct photosynthetic electrons from PQ to PSI

To characterize the effect of introducing *OmcS*, we used two site-specific photosynthetic electron transfer inhibitors 3-(3,4dichlorophenyl)-1,1-dimethylurea (DCMU) and 2,5-dibromo-3-methyl-6-isopropylbenzoquinone (DBMIB), respectively [37] (Fig. 1).

**Fig. 3:**
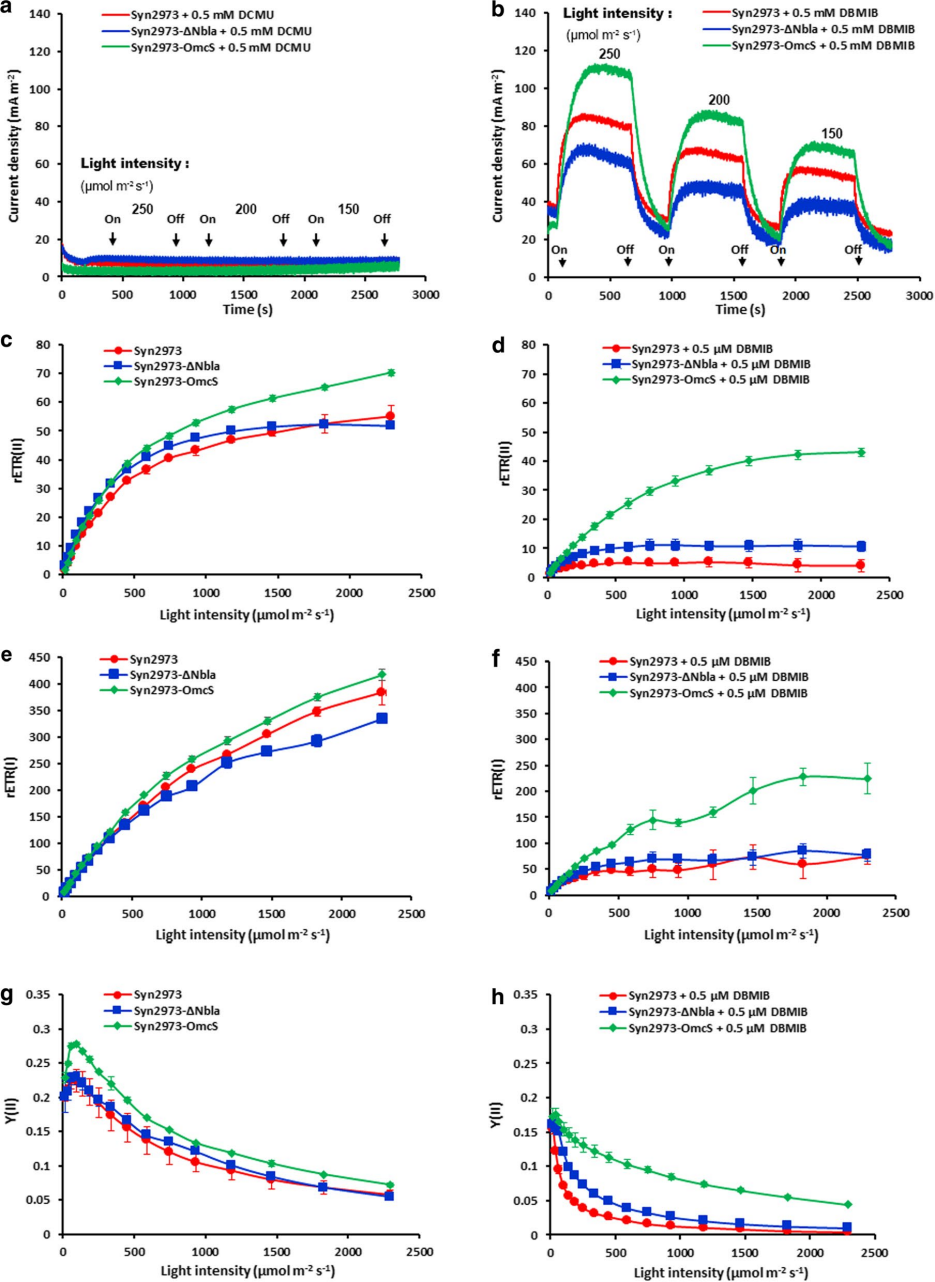

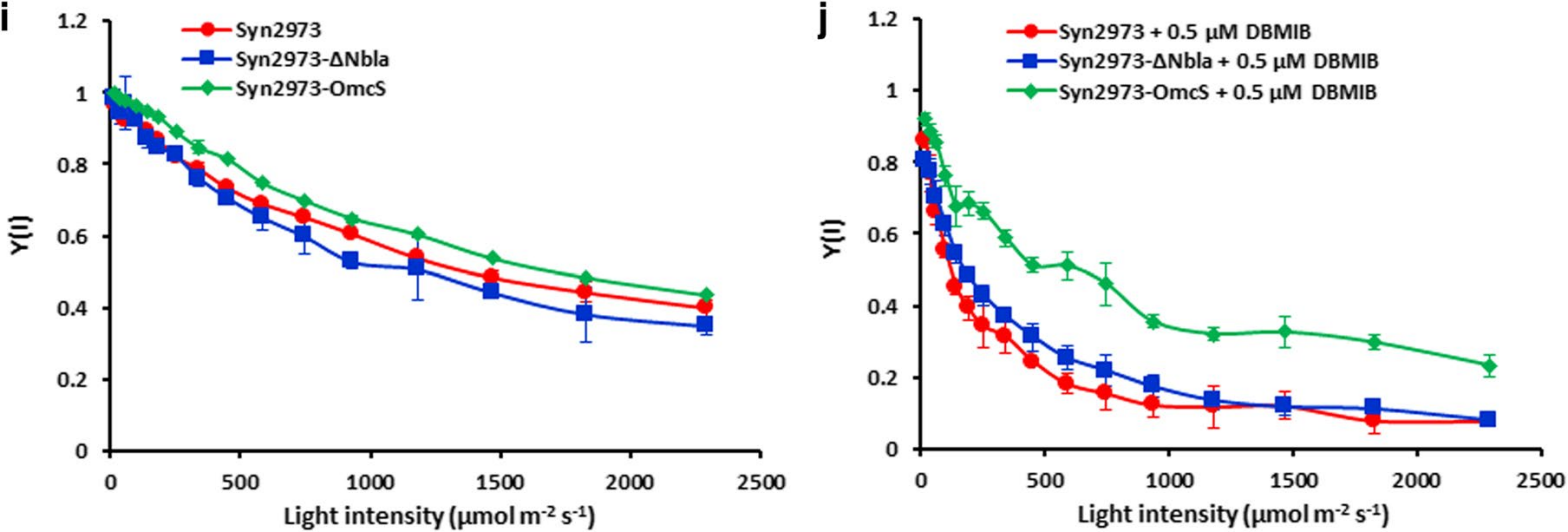
Effects of the introduced OmcS on photocurrent and photochemical activity. **a**, **b** Photocurrent response to site-specific photosynthesis inhibitor DCMU and DBMIB, respectively, under varied light intensity. **c**–**j** Photochemical activity assay of PSII and PSI with or without inhibitor DBMIB

DCMU is a PSII inhibitor, which blocks the PQ binding site of PSII [37, 38]. We used DCMU to test if OmcS can direct electrons from PSII (Fig. 3a). If positive, OmcS may bypass the DCMU inhibitor between PSII and PQ, and we should be able to detect an increased photocurrent generation from strain Syn2973-OmcS when supplemented with 0.5 mM DCMU, a concentration which is sufficient to completely block the electron transfer from PSII to PQ [37]. However, we did not observe increased photocurrent in strain Syn2973-OmcS, nor in its control strains Syn2973 and Syn2973-ΔNbla, when supplemented with 0.5 mM DCMU (Fig. 3a). This demonstrated that OmcS is not able to direct the electrons from PSII.

DBMIB is a cyt*b6f* inhibitor, which blocks the PQ binding site of cyt*b6f* complex [37]. We used DBMIB to test if OmcS can direct electrons from PQ pool (Fig. 1). Similarly, if positive, we should be able to detect an increased photocurrent generation from strain Syn2973-OmcS compared to its control strains Syn2973 and Syn2973-ΔNbla when supplemented with 0.5 mM DBMIB, a concentration which is sufficient to completely block the electron transfer from PQ to cyt*b6f* [37]. As Fig. 3b showed, under different light intensities (250, 200, and 150 μmol photons m^−2^ s^−1^), the photocurrent generated by strain Syn2973-OmcS treated with DBMIB was approximately onefold or 50% higher than that of strains Syn2973-ΔNbla and Syn2973 treated with 0.5 mM DBMIB, respectively. The increase of photocurrent by strain Syn2973-OmcS treated with DBMIB, together with the negative results with DCMU, demonstrated that OmcS is able to direct the electrons from PQ.

Furthermore, we wanted to know where the electrons that OmcS directed from PQ went. The LET transfers photosynthetic electron from PSII to PSI through PQ and cyt*b6f* [22, 34] (Fig. 1). To test if the electrons that OmcS directed from PQ went to PSI, we investigated the chlorophyll fluorescence kinetics of PSII and the energy conversion efficiency of PSI when supplemented with DBMIB, which blocks electrons transfer from PQ to cyt*b6f* [37] (Fig. 3c–j). We could not observe the light response curves of the relative electron transport rate of PSII rETR(II) and of PSI rETR(I) in strains Syn2973-ΔNbla and Syn2973 when supplemented with 0.5 μM DBMIB, suggesting that 0.5 μM DBMIB completely blocked the photosynthetic activity of PSII and PSI (Fig. 3d and f). Whereas for strain Syn2973-OmcS, we did observe clearly the light response curve of both rETR(II) and rETR(I), and OmcS can rescue more than 50% of the inhibition that DBMIB exerted on rETR(II) and rETR(I) (Fig. 3c–f). The effective quantum yield of PSII Y(II) and PSI Y(I) also showed almost the same tendency with that of rETR(II) and rETR(I) (Fig. 3h and j). Both Y(II) and Y(I) in strains 2973-ΔNbla and Syn2973 could not be observed upon the addition of 0.5 μM DBMIB, while OmcS could rescue the inhibition of DBMIB on Y(II) and Y(I) in Syn2973-OmcS strain (Fig. 3g–j). These data demonstrated that OmcS can bypass the inhibition of DBMIB via directing electrons from PQ to PSI.

### Cells with OmcS show an increased level of ATP and NADH

In cyanobacteria, LET generates ATP and NADPH at a fixed ratio, CET generates ATP without net NADPH formation, while respiratory electron transfer (RET) generates ATP at the expense of NADH [19, 22, 32, 34] (Fig. 1). Thus, the profile of intracellular concentrations of ATP, NADPH and NADH may reflect the activity of LET, CET, and RET. We therefore quantitatively determined the intracellular concentrations of ATP, NADPH and NADH in strains Syn2973, Syn2973-ΔNbla, and Syn2973-OmcS (Table 1). Compared with its control strain Syn2973-ΔNbla, the level of ATP and NADH in strain Syn2973-OmcS increased by 37% and 58%, respectively, while the level of NADPH only increased by 3% (Table 1). The remarkably increased ratio of ATP/ADP and NADH/NAD^+^, with the slightly increased ratio of NADPH/NADP^+^ in Syn2973-OmcS strain, further confirmed our initial hypothesis. The increased ATP level in strain Syn2973-OmcS is not positively correlated with its NADPH level. This indicates the increased ATP production in strain Syn2973-OmcS is not all from LET, as LET generates ATP and NADPH in a fixed ratio. We also observed that the increased ATP level in strain Syn2973-OmcS is associated with an increased NADH level. This indicates the increased ATP production in strain Syn2973-OmcS is not from RET either, as RET generates ATP at the expense of NADH. It is conceivable that the increased ATP production in strain Syn2973-OmcS saved the NADH from RET that otherwise would be used to generate ATP, thus resulted in an increased NADH level.

**Table 1:** Measurement of ATP, ADP, NADH, NAD^+^, NADPH and NADP^+^ in strains during exponential growth phase

Strains	ATP	ADP	ATP/ADP	NADH	NAD^+^	NADH/NAD^+^	NADPH	NADP^+^	NADPH/NADP^+^
Syn2973	626.1 ± 24.6	327.1 ± 2.2	1.92 ± 0.09	32.1 ± 2.6	72.6 ± 1.9	0.44 ± 0.05	47.6 ± 4.0	153.3 ± 13.6	0.32 ± 0.05
Syn2973-ΔNbla	605.3 ± 25.7	353.7 ± 23.8	1.71 ± 0.09	39.0 ± 1.0	100.5 ± 2.3	0.39 ± 0.02	50.8 ± 1.1	151.2 ± 7.8	0.34 ± 0.02
Syn2973-OmcS	828.3 ± 13.9	222.6 ± 6.3	3.72 ± 0.04	61.7 ± 2.1	67.4 ± 5.2	0.92 ± 0.10	52.5 ± 1.0	143.1 ± 1.8	0.37 ± 0.01
Syn2973-Ldh	548.1 ± 21.0	296.9 ± 0.9	1.85 ± 0.07	33.7 ± 2.3	100.8 ± 5.8	0.34 ± 0.04	44.7 ± 3.4	134.7 ± 5.0	0.33 ± 0.03
Syn2973-LdhΔGlcD1	541.7 ± 28.0	334.5 ± 15.9	1.62 ± 0.01	27.2 ± 1.4	83.9 ± 2.4	0.32 ± 0.01	40.7 ± 0.4	154.7 ± 0.9	0.26 ± 0.01
Syn2973-LdhOmcS	699.0 ± 12.9	253.4 ± 21.1	2.78 ± 0.28	39.4 ± 1.1	94.0 ± 3.7	0.42 ± 0.01	46.9 ± 0.6	149.0 ± 1.6	0.32 ± 0.01
Syn2973-LdhOmcS-2NP	769.0 ± 19.8	219.9 ± 14.6	3.52 ± 0.32	40.1 ± 2.2	99.7 ± 6.8	0.40 ± 0.01	47.8 ± 4.2	136.8 ± 3.9	0.35 ± 0.04

The data are mean of three independent measurements (mean ± SD; pmol OD_730_^−1^)

### Cells with OmcS can supply sufficient NADH for d-lactate production

To test if the introduced OmcS can provide sufficient NADH for chemical production in cyanobacteria, we used the production of lactate, which requires NADH, as a model. We introduced an NADH-dependent d-lactate dehydrogenase (d-Ldh) which converts pyruvate into d-lactate at the expense of NADH (Fig. 1), as an extra NADH-consumption pathway to introduce perturbations on intracellular NADH levels.

We inserted *omcS* into the *glcD1* locus of the d-lactate-producing strain Syn2973-Ldh, which carries a d-Ldh from *Lactobacillus delbrueckii* ATCC 11,842 in the *nbla* locus [26, 39]. *glcD1* encodes a glycolate dehydrogenase involving in glycolate metabolism in a cyanobacterium and disrupting *glcD* resulted in a high-CO_2_-requirement phenotype [40]. We designated the resulting strain carrying *ldh* and *omcS* as Syn2973-LdhOmcS [41]. Then we constructed Syn2973*-*LdhΔGlcD1 strain as the control strain of Syn2973-LdhOmcS by replacing the *glcD1* with kanamycin on the basis of strain Syn2973*-*Ldh (Fig. 2a). PCR and sequencing verified the integration of genes and full chromosome segregation (Fig. 2b). RT-PCR showed the introduced *omcS* and *ldh* in strains Syn2973-OmcS, Syn2973-LdhΔGlcD1 and Syn2973-LdhOmcS were transcribed, respectively (Fig. 2c), confirming the expression of genes *omcS* and *ldh* in these strains. Since the expression of genes *ldh* and *omcS* were under control of strong promoter P_cpc560_ [42] and weak promoter P_rbcL200_, respectively, the expression level of *ldh* gene was up to approximately 34-fold of that of *omcS* gene (Fig. 2d, e).

**Fig. 4:**
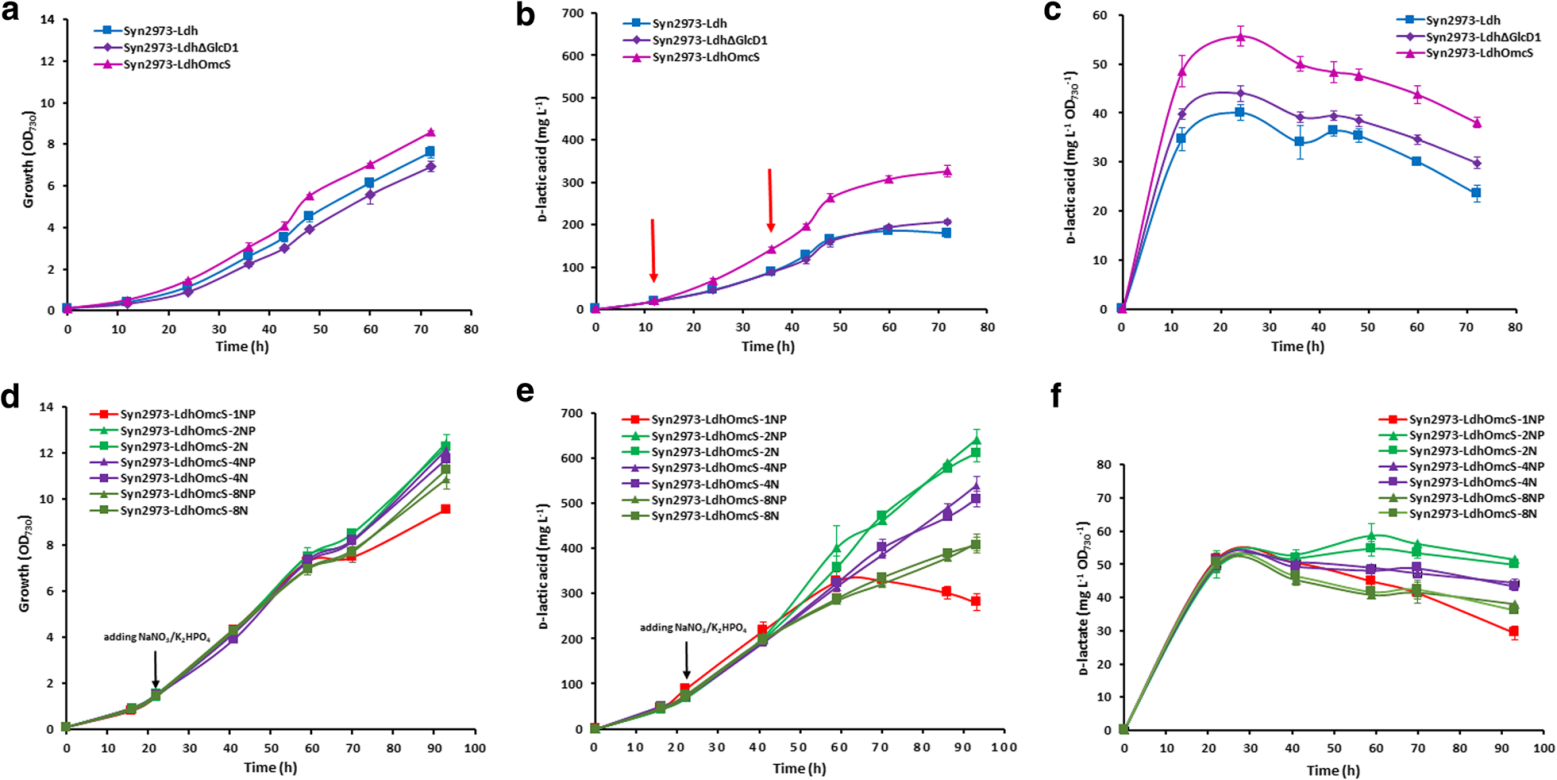
Growth curves and d-lactate production of corresponding strains. **a**–**c** Under normal conditions. **d**–**f** Under extra nitrogen and phosphate supplemented conditions. Syn2973-LdhOmcS-1NP: the strain Syn2973-LdhOmcS grew in the common BG11 medium, Syn2973-LdhOmcS-2NP-8NP: the strain Syn2973-LdhOmcS grew in BG11 medium with twofold to eightfold concentration of nitrate and phosphate, Syn2973-LdhOmcS-2 N-8 N: the strain Syn2973-LdhOmcS grew in BG11 medium with twofold to eightfold concentration of nitrate. Values are average of three independent measurements; Error bars indicate the standard deviation (SD); if error bars are invisible, they are small than the data point symbol

First, we determined the d-lactate production by strains Syn2973, Syn2973-OmcS, Syn2973-Ldh, Syn2973-LdhΔGlcD1 and Syn2973-LdhOmcS (Fig. 4). As expected, wild-type Syn2973 and Syn2973-OmcS did not accumulate d-lactate, while mutant strain Syn2973-Ldh produced 185.5 ± 7.1 mg L^−1^ d-lactate. Strain Syn2973-LdhOmcS produced up to 326.4 ± 14.1 mg L^−1^ d-lactate, while the control strain Syn2973-LdhΔGlcD1, without *omcS* in *glcD1* locus, produced 206.6 ± 4.2 mg L^−1^ d-lactate (Fig. 4b). These data suggest that the introduced OmcS facilitated the production of d-lactate in cyanobacterial strain Syn2973-LdhOmcS.

Notably, strain Syn2973-LdhOmcS grew faster than the Syn2973-Ldh and Syn2973-LdhΔGlcD1 (Fig. 4a). The OD_730_ of Syn2973-LdhOmcS was 23.7% higher than its control strain Syn2973-LdhΔGlcD1 in 60 h. To exclude the effect of the improved biomass growth rate, we plotted d-lactate production per OD in Fig. 4c. During the whole fermentation period, the specific d-lactate production ability of strain Syn2973-LdhOmcS increased by 23–48%, compared with the control strain Syn2973-LdhΔGlcD1. The data demonstrated that cyanobacterial cells with OmcS favors the NADH-dependent production of d-lactate.

Then, we determined the level of ATP, NADPH, and NADH in d-lactate producing strains Syn2973-Ldh, Syn2973-LdhΔGlcD1 and Syn2973-LdhOmcS (Table 1). Overall, the expression of d-Ldh in cyanobacterial cells reduced intracellular ATP levels (Table 1). This is very likely due to protein burden as d-Ldh can be expressed to about 10% of the soluble proteins of the cyanobacterial cells (Fig. 2e). The ATP level and the ratio of ATP/ADP, the NADPH level and the ratio of NADPH/NADP^+^ of strain Syn2973-LdhOmcS were all higher than that of strain Syn2973-LdhΔGlcD1. However, the degree of improvement on ATP level and ATP/ADP is more prominent than that on NADPH level and NADPH/NADP^+^. This suggests that the CET and LET in cells with OmcS are all improved by the additional electrons flow directed from PQ to PSI, but the contribution from the improvement of CET (producing ATP only) is higher than that from LET (producing NADPH and ATP). This is also the case if comparing the ATP (ATP/ADP) and NADPH (NADPH/NADP^+^) levels of strain Syn2973-OmcS and its controls strains.

Moreover, the NADH concentrations were determined in d-lactate producing strains Syn2973-Ldh, Syn2973-LdhΔGlcD1 and Syn2973-LdhOmcS (Table 1). As expected, the NADH levels of all d-lactate-producing strains Syn2973-Ldh, Syn2973-LdhΔGlcD1 and Syn2973-LdhOmcS were lower than that of its controls strains, due to the need of consuming NADH for d-lactate production. However, although strain Syn2973-LdhOmcS produced 76% more d-lactate than that of strain Syn2973-Ldh, the NADH level and the NADH/NAD^+^ of strain Syn2973-LdhOmcS were significantly higher than those of Syn2973-Ldh and Syn2973-LdhΔGlcD1 (Table 1). Even under the circumstance of providing ATP for the expression of heterogeneous proteins and providing NADH for the production of d-lactate, strain Syn2973-LdhOmcS was still able to maintain a 29% higher ATP level than that of strain Syn2973-LdhΔGlcD1 and exhibit a higher growth rate. This further demonstrated that strain Syn2973-LdhOmcS has obtained a stronger ATP generating ability which is independent of the generation of either NADPH or NADH.

Since strain Syn2973-LdhOmcS grows faster than its control strains, we wanted to further test whether we can observe a similar effect at more nutritious conditions and whether the introduced OmcS can provide sufficient NADH for more d-lactate production. To this end, we added more nitrate and phosphate in BG11 medium to achieve higher production of biomass and production of d-lactate. Upon addition of twofold nitrate and phosphate, the growth of Syn2973-LdhOmcS cells increased up to 32%, while d-lactate production nearly doubled, from 328.8 ± 1.6 to 641.4 ± 23.5 mg L^−1^ (Fig. 4d, e). Moreover, the specific d-lactate production ability of Syn2973-LdhOmcS upon addition of twofold nitrate and phosphate also increased (Fig. 4f). Subsequently, we determined the intracellular concentration of ATP, NADPH and NADH in Syn2973-LdhOmcS supplemented with twofold nitrate and phosphate (designated as Syn2973-LdhOmcS-2NP, Table 1). Although strain Syn2973-LdhOmcS-2NP grew to a higher OD and produced one-fold higher concentration of lactate than strain Syn2973-LdhOmcS did, the intracellular profile of ATP, ATP/ADP, NADPH, NADPH/NADP^+^ and NADH and NADH/NAD^+^ in strain Syn2973-LdhOmcS-2NP was similar to that of strain Syn2973-LdhOmcS (Table 1). Compared with strain Syn2973-LdhΔGlcD1, strain Syn2973-LdhOmcS-2NP also showed a significantly increased ATP, NADH and NADPH concentrations, indicating cells with OmcS can supply sufficient NADH for the production of d-lactate.

To exclude the effect of improved biomass growth rate (improved photosynthesis efficiency) on the increased lactate production, lactate production per OD was plotted in Fig. 4. As the improved cell growth rate is directly correlated with the improved photosynthesis efficiency, the significantly increased specific d-lactate production ability (d-lactate production per OD, Fig. 4c and f) indicated that the increased NADH level is the main factor contributing to the increased d-lactate production and the redirection of electrons from PQ to PSI in cells with OmcS can provide additional NADH for more d-lactate production.

### Cells with OmcS show an increased photochemical activity of PSII and PSI

**Fig. 5:**
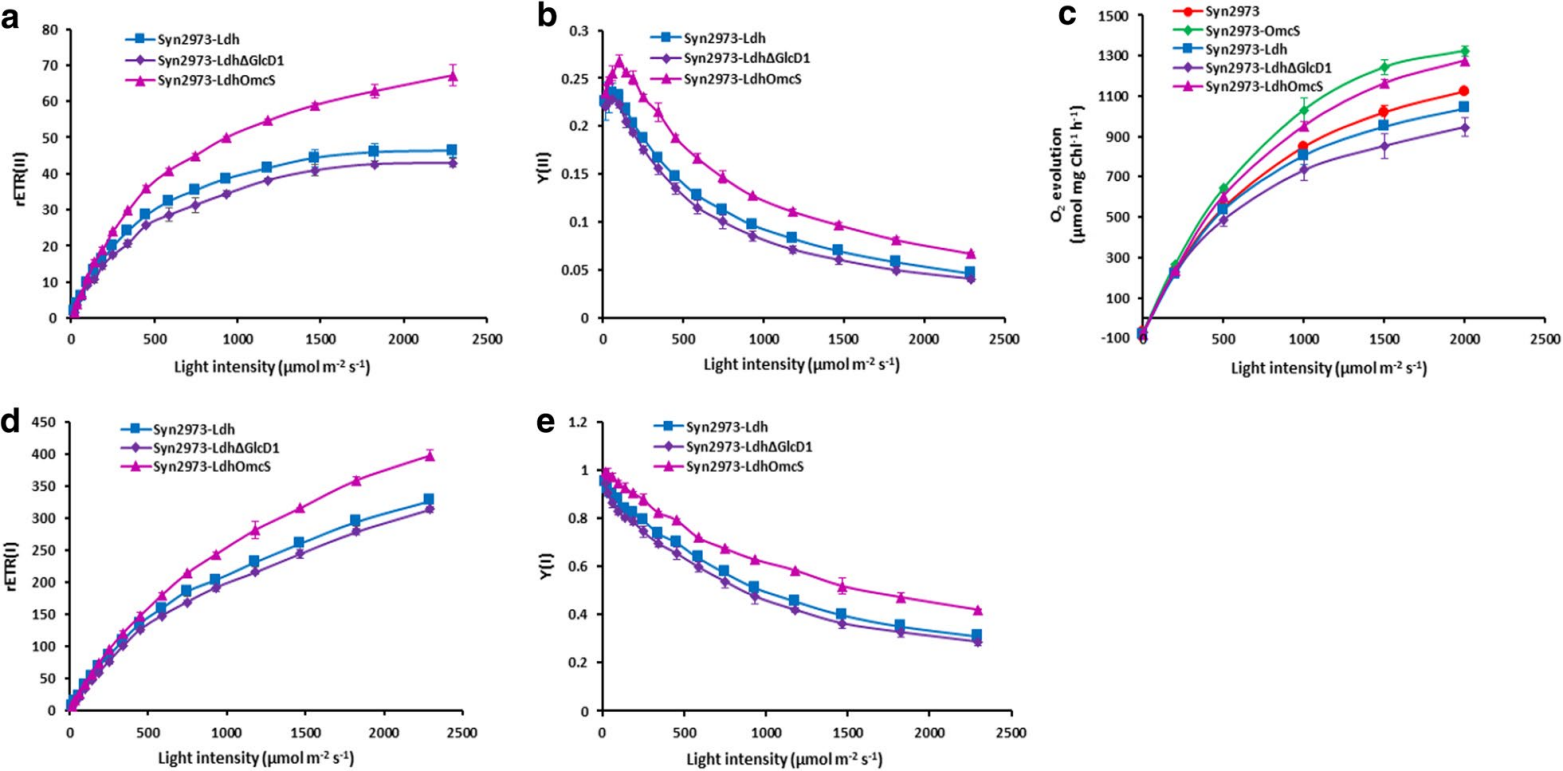
Photosynthetic physiological analyses of the PSII and PSI in d-lactate producing strains. **a** Light response curve of rETR(II) of PSII. **b** Light response curve of Y(II) of PSII. **c** Oxygen evolution of the PSII under varied light intensities (from 0 to 2000 μmol photons m^−2^ s^−1^). **d** Light response curve of rETR(I) of PSI. **e** Light response curve of Y(I) of PSII. Prior to measurements, cells in the exponential phase were wash and re-suspended in fresh BG11 medium containing 50 mM NaHCO_3_. The experimental point are averages of three independent measurements; Error bars indicate the standard deviation (SD); if error bars are invisible, they are small than the data point symbol. Abbreviations: rETR(II), relative electron transport rate of PSII; Y(II), effect quantum yield of PSII. Error bars indicate the standard deviation (SD); if error bars are invisible, they are smaller than the label of the diagram

To investigate the effect of introducing OmcS on the activity of photosynthetic systems (PSII and PSI), we determined the light response curves of rETR(II) of these strains. As shown in Figs. 3c and 5a, rETR(II) of *omcS* carrying strains Syn2973-OmcS and Syn2973-LdhOmcS were significantly higher than that of their respective control strains Syn2973-ΔNbla (Fig. 3c) and Syn2973-LdhΔGlcD1 (Fig. 5a), and even higher than that of the wild-type Syn2973, under different illumination intensities. The related electron transport rate of PSII (rETR (II)) of the strain Syn2973-LdhΔGlcD1 was 36.4% and 31.2% lower than strains Syn2973-OmcS and Syn2973-LdhOmcS under semi-light saturation light intensity (932 μmol photons m^−2^ s^−1^), respectively. Besides, similar results were observed in Y(II) (Figs. 3g and 5b). The Y(II) of strain Syn2973-LdhOmcS was 49.1% and 64.6% higher than that of strain Syn2973-LdhΔGlcD1 under 932 μmol photons m^−2^ s^−1^ and 1826 μmol photons m^−2^ s^−1^, respectively (Fig. 5b).

Moreover, the rate of O_2_ evolution of these strains was determined under different illumination intensity (from 0 to 2000 μmol photons m^−2^ s^−1^). As shown in Fig. 5c, the O_2_ evolution rate of strain Syn2973-LdhOmcS was much faster than that of the control strain Syn2973-LdhΔGlcD1 and even the wild-type Syn2973 under the same light intensity. The O_2_ evolution rate of strain Syn2973-LdhOmcS was 1.24-fold that of Syn2973-LdhΔGlcD1 under 500 μmol photons m^−2^ s^−1^. Similarly, the O_2_ evolution rate of strain Syn2973-OmcS was 1.18-fold that of wild-type Syn2973 (Fig. 5c).

The above data demonstrated that the rETR(II), Y(II) and O_2_ evolution rate of strain Syn2973-LdhOmcS were significantly higher than that of strains Syn2973-LdhΔGlcD1 and Syn2973-Ldh, while the rETR(II), Y(II) and O_2_ evolution rate of strain Syn2973-LdhΔGlcD1 were lower than those of Syn2973-Ldh (Fig. 5a–c). These meant it was the introduced OmcS that improved photosynthetic activity of PSII, rather than the knockout of *glcD1* [40] did.

Furthermore, the effects of the introduced OmcS on PSI were demonstrated. rETR(I) of *omcS* carrying strains Syn2973-OmcS and Syn2973-LdhOmcS were significantly higher than that of their respective control strains Syn2973-ΔNbla and Syn2973-LdhΔGlcD1 under various illumination intensity (Figs. 3e and i, 5d and e). The rETR(I) of strains Syn2973-OmcS and Syn2973-LdhOmcS were 34.4% and 26.7% higher than that of strain Syn2973-LdhΔGlcD1 under 932 μmol photons m^−2^ s^−1^, respectively. Besides rETR(I), similar results were observed in Y(I) (Figs. 3i and 5e). The Y(I) of strain Syn2973-LdhOmcS was 18.3% and 32.1% higher than the strain Syn2973-LdhΔGlcD1 under cultured light intensity (342 μmol photons m^−2^ s^−1^) and 932 μmol photons m^−2^ s^−1^, respectively. These results demonstrated that the photochemical activity of PSI was increased in cells with OmcS.

### Introduction of OmcS upregulates the expression of genes encoding key enzymes of photosynthesis

To gain insights into the mechanisms how introducing OmcS augments photosynthetic electron transfer to generate more ATP and contributes to providing sufficient NADH, we analyzed the transcriptome of d-lactate production strains with or without OmcS sampled at two time points during rapid d-lactate production period (Figs. 1 and 4b). Because the growth and photosynthetic activity of Syn2973-LdhΔGlcD1 were significantly weaker than that of Syn2973-Ldh (Figs. 4a and 5), we used strain Syn2973-Ldh as control for transcriptome analysis of strain Syn2973-LdhOmcS (Fig. 1, Additional file 1: Table S1–S4).

First, we analyzed the effect of introducing OmcS on the transcription of genes encoding key enzymes of photosynthesis antenna, PSII, PSI, cyt*b6f* complex and photosynthetic electron transport (Fig. 1, Additional file 1: Table S2 and S3). Phycobilisome (PBS), which is composed of allophycocyanin (AP), phycocyanin (PC)/phycoerythrocyanine (PEC) and phycoerythrin (PE) is the main light-harvesting complex in cyanobacteria [43]. Compared to strain Syn2973-Ldh, most subunits of AP, PC/PEC and PE, especially ApcE and ApcD subunits of AP, the terminal energy emitters in the PBS core, which funnels light energy directly to PSII and PSI [43] in Syn2973-LdhOmcS strain, were significantly upregulated (Fig. 1, Additional file 1: Table S2). This indicates both PSII and PSI can absorb more light. In addition, the reaction center subunit D1 protein, PSII core antenna proteins CP43, CP47, Psb27, Psb28, PsbV and oxygen-evolving enhancer protein 1 (MSP) [43, 44] in strain Syn2973-LdhOmcS also significantly upregulated (Fig. 1, Additional file 1: Table S3). Moreover, some core proteins of PSI including PsaC, PsaD, PsaF and PsaL subunits in strain Syn2973-LdhOmcS were significantly upregulated (Fig. 1, Additional file 1: Table S3), of which PsaL subunit is in charge of multimer form formation of PSI under high light [45, 46]. Interestingly, cyt*b6f* and PC involved in photosynthetic electron transport and ferredoxin-NADP^+^ reductase (FNR) involved in NADPH formation were also upregulated (Fig. 1, Additional file 1: Table S3). This further supported the increased photosynthetic activity of PSII and PSI in Syn2973-LdhOmcS strain.

Secondly, we analyzed the transcription of key enzymes involved in oxidative phosphorylation, through which ATP is synthetized (Fig. 1, Additional file 1: Table S4). Compared to strain Syn2973-Ldh, the introduced OmcS significantly upregulated the expression of NAD(P)H-quinone oxidoreductase complex (NDH), cytochrome bd-quinol oxidase complex (Cybd) and F-type ATPase, whereas the expression of cytochrome c oxidase complex (CoxC) in strain Syn2973-LdhOmcS significantly downregulated at both time points. F-type ATPase is in charge of ATP synthesis and the significantly upregulated almost all subunits of both F_0_ and F_1_ of ATPase were consistent with the increased ATP level in strain Syn2973-LdhOmcS.

NDH is involved in NDH-CET and respiration in cyanobacteria [34, 47, 48]. OmcS significantly upregulates NdhA, NdhD, NdhE, NdhF, NdhG, NdhE, NdhJ, NdhL, NdhN and NdhI subunits of NDH, some of which are core subunits and some of them potentially accepts electrons from PSI in cyanobacteria [47–49]. CoxC competes electrons with PSI and is in charge of RET under light condition, while Cybd is the oxidase which accepts electrons directly from PQ from PSII and does not pump protons across the membrane [50]. Flux balance analysis also showed that stimulating CET enhanced the Cybd flux, while decreased CoxC flux [32]. The downregulated CoxC indicated the activity of RET is decreased and the upregulated Cybd was consistent with the increased CET. These data indicated that the introduced OmcS contributed to ATP synthesis and the increased ATP resulted from the upregulated CET rather than from RET.

Generally, there are mainly two types of CET in oxygenic photosynthetic organisms. One is the PGR5-dependent CET in which the electrons are transferred from Fd of PSI to cyt*b6f*, PC and then back to PSI [20] (Fig. 1, the grey line). The other is the NDH-dependent CET in which the electrons are transferred from FNR-NADPH of PSI to NDH, PQ to cyt*b6f*, PC, and then back to PSI (Fig. 1, the red line) [7, 20, 31, 48]. It is generally considered that the PGR5-dependent CET is dominant in plant and algae, while NDH-dependent CET is dominant in cyanobacteria, although PGR5-dependent CET was recently also found in cyanobacteria [7, 20, 31, 48]. In this work, the significantly upregulated NDH and FNR and downregulated Fd indicated the activated CET in strain Syn2973-LdhOmcS is NDH-dependent.

Moreover, we analyzed the transcription pattern of genes encoding key enzymes involving in central metabolism. We found the expression of genes involved in the Calvin cycle, glycolysis, TCA cycle and pentose phosphate pathway in strain Syn2973-LdhOmcS were generally upregulated (Fig. 1, Additional file 1: Table S4). Key genes of Calvin cycle including *rbcL* and *rbcS* of Rubisco, *rpe,* and *prk* were upregulated at least threefold. *pgk* and *gapA* of glycolysis pathway were upregulated more than twofold. In TCA cycle, *icd* was upregulated by twofold and *ogdh*/*sucA* was upregulated at least eightfold. *fbp*, *glpX*, *pgi*, *tktA*/*tktB* of pentose phosphate pathway were significantly upregulated, in which, *fbp*, *glpX*, *tktA*/*tktB* were upregulated at least eightfold. The data indicated that the introduced OmcS stimulated Calvin cycle, glycolysis, TCA cycle, and pentose phosphate pathway in strain Syn2973-LdhOmcS.

To our understanding, the comparative transcriptome analyses indicated that the introduced OmcS led to three effects: (1) improves CET, which increased ATP production; (2) improve LET, which increased both ATP and NADPH production; (3) represses RET, which saved the NADH that otherwise will be respired to generate ATP, thus leading to an increase NADH level. Collectively, the combination of these three effects is shown as the increased photosynthesis efficiency, increased cell growth and increased production of d-lactate. Moreover, the improved photosynthesis can provide more carbon precursors contributing to d-lactate production. Comparative transcriptome analyses coincide well with physiological and biochemical analysis. It provides hints for us to understand why the ATP and NADH levels are increased in cells with OmcS.

## Discussion

In oxygenic photosynthetic organisms, light energy converts to chemical energy ATP and NADPH via photosynthetic electron transfer, then cells consume ATP and NADPH for carbon fixation and metabolism (Fig. 1). When the chemical energy, especially NADPH exceeds the energy consumption for cell growth and metabolism, it is toxic to the photosynthetic membrane and results in photoinhibition and even photodamage [23]. Generally, LET from PSII to PSI is in charge of the generation of ATP and NADPH at a fixed ratio, while CET around PSI without net NADPH production plays important roles in regulating the ratio of ATP and NADPH to accumulating to environmental challenges [20, 31, 48]. Therefore, enhancing CET to regulating the ratio of ATP and NADPH to meet metabolic demand is essential for efficient photosynthesis. In this work, we designed, developed, and tested an efficient strategy to enhance CET to regulate the ratio of ATP and NADPH to meet metabolic demand in cyanobacterium Syn2973. The core idea of the strategy is based on the hypothesis that redirecting excess electrons from PQ via an electron carrier OmcS can drive PQ accepting more electrons from PSI and enhance CET.

Previous reports have shown that exoelectrogens possess the ability to transport electrons extracellularly, and this was ascribed to the presence of numerous c-type outer membrane cytochromes (Omc) [51]. Among them, electron transport protein OmcS plays a key role in extracellular electron transport (EET) in *Geobacter* sp. Previous work showed that the introduction of *omcS* in Syn7942 significantly enhanced its EET ability when an extracellular electrode is available. That work demonstrated OmcS was expressed as a soluble protein and can direct electrons in strain Syn7942 [27, 37]. However, the binding site of OmcS and its effects on biochemical and photosynthetic physiology remain unknown. Here, based on the analysis of electrochemistry and photosynthetic physiology with site-specific photosynthetic electron transfer inhibitors, we demonstrated that OmcS directs electrons from PQ to PSI to stimulate the CET of cyanobacteria. Various cyanobacteria possess the EET activity to dissipate excess electrons in response to illumination and a more reduced PQ pool contributes to EET [27, 37, 52]. Thus, channeling the extra electrons from PQ to stimulate CET via OmcS, rather than channeling into EET to be wasted, is an effective way to increase the efficiency of utilizing photons.

To our understanding, the increased PS activity was due to the expression of OmcS which redirected the excess electrons from PQ to PSI and stimulated CET as well as LET. We argue that such an alternative electron flow mediated by OmcS might have increased the overall electron flow efficiency, which cannot be simply achieved by only overexpressing the proteins involved in native photosystem machineries as it is usually stringently regulated. We think overexpressing other electron-transfer proteins with similar electrochemical characteristics of Omcs might exert a similar effect, and this can be tested in future research.

In cyanobacteria, three main electron transfer chains exist, i.e. LET, CET, and RET. The activities of these electron transfer chains determine the intracellular level of ATP, NADPH and NADH. Proteins involved in LET, CET and part of RET are located in the thylakoid membrane and PQ is shared by all three electron transport chains [19, 22, 32, 34] (Fig. 1). All three electron transfer chains generate ATP but with different features. LET from PSII to PSI generates ATP and NADPH in a fixed ratio. As an auxiliary, CET generates ATP, but without net NADPH formation, by cycling electrons from PSI back to the PQ pool. RET generates ATP from NADH, which is mainly derived from catabolic metabolism.

In this work, OmcS as an electron carrier channels excess photosynthetic electrons from PQ to PSI and consequently, stimulates CET as well as drives LET. LET generates ATP and NADPH at a fixed ratio, while CET cycles electrons from PSI back to PQ pool and generates ATP without net NADPH production. The main ATP and NADPH was used for carbon fixation. In this work, the growth of the OmcS-expressing strain was better than its control strain, indicating OmcS-expressing strain consumed more ATP and NADPH for carbon fixation than its control strain, which is also a consequence of the increased photosynthesis. The sum of the increased LET (increase ATP and NADPH proportionally), increased CET (increase ATP only) and increased biomass production (consume ATP and NADPH proportionally) reflected as the significantly increased ATP concentration but only slightly increased NADPH concentration (an increased ratio of ATP/NADPH overall) that we observed. This explains why photosynthesis increased but the photosynthesis NADPH remains constant between these strains. Therefore, an increased ratio of ATP/NADPH in OmcS-expressing strain is due to an increased CET.

Our data from photosynthetic activity analysis (PSII and PSI) and comparable transcriptome analysis demonstrated that OmcS is functional as an electron transfer bypass channeling electrons from PQ pool to PSI. The introduction of OmcS improves CET and LET but represses RET. The improved CET results in increased ATP, the improved LET results in increased ATP and NADPH, while repressed RET results in increased NADH. Therefore, in this work, the level of ATP and NADH increased, but the level of NADPH did not decrease. The intracellular NADH level of the d-lactate producing strains sharply decreased, but there are no significant difference among the four d-lactate producing strains with or without OmcS. This further demonstrates that the increased ATP levels may repress RET and thus provide sufficient NADH for d-lactate production in strains with OmcS.

NADH is the major reducing equivalent used for lactate production. One exception is a Ldh from *B. subtilis*, which is capable of using both NADPH and NADH. Kinetic assays shown that the Km value of Ldh from *B. subtilis* for NADPH is 0.288 mM, while its Km for NADH is only 0.013 mM [53]. It means the affinity of this Ldh to NADPH is very low, that limits its application in NADPH-rich microbes, such as cyanobacteria (Li et al., 2015; Varman et al., 2013). Switching the cofactor preference from NADH into NADPH of Ldh though directed-mutagenesis (Angermayr, 2014; Li et al., 2015) or expression of a soluble transhydrogenase to convert NADPH into NADH (Angermayr et al., 2012; Niederholtmeyer et al., 2010) did increase the production of lactate in NADPH-rich cells. To date, the highest lactate production is 1.84 g L^−1^ in *Synechocystis* sp. PCC 6803 with a long fermentation period (4 weeks) [54] and the maximal productivity is 221 mg L^−1^ day^−1^ (amount to 2.45 mM day^−1^) using an NADPH-dependent lactate dehydrogenase in Syn7942 [9] (Li et al., 2015). Although it might help to switch the cofactor preference of the Ldh from NADH to NADPH, we should note that the employment of these enzymes often resulted in impairment of growth. The maximal productivity of 2.54 mM day^−1^ in this work is comparable to the reported highest productivity of 2.45 mM day^−1^ by engineered cyanobacteria. Moreover, the lactate production and the maximal productivity was increased one fold after supplemented with sufficient nitrate and phosphate, indicating the strategy of directing electrons from PQ pool to PSI in cells with OmcS can provide more NADH for more lactate production without growth defect.

Keeping the dynamic balance of NADH would have a great impact on the metabolic engineering of phototrophic organisms. Compared with other strategies, such as using NADPH-dependent enzymes or introducing a transhydrogenase to convert the NADPH into NADH, the novelty of this research is directing electrons from PQ pool to PSI. This augmented the photosynthetic electron transfer, generating more ATP from light energy, so as to direct the NADH from being consumed in RET for chemicals production. The strategy described in this study could be further tested in other oxygenic, photoautotrophic microorganisms and higher plants to check whether similar improvements in photosynthesis or chemicals production could be achieved.

## Conclusions

In this work, we developed a new strategy to provide sufficient NADH from excess photosynthetic electrons for the production of d-lactate in phototrophic microbes cyanobacterium Syn2973. The core idea of this strategy was to augment photosynthetic electron transfer to generate more ATP by introducing OmcS, saving NADH that otherwise will be consumed for ATP production through the respiratory electron transfer chain. Our data confirmed that the introduced OmcS channels excess photosynthetic electrons from the reduced PQ site to PSI to stimulate CET. As a result, more ATP was generated, while RET was repressed. This strategy did provide sufficient NADH for d-lactate production. Moreover, directing the excess photosynthetic electrons via the introduced OmcS also increased photosynthesis efficiency due to regulating LET and CET.

## Methods

### Strains and growth conditions

The strains used in this study were listed in Additional file 1: Table S5. For the plasmid construction, *Escherichia coli* (*E. coli*) strain DH5α was cultured at 37 °C in Luria–Bertani (LB) broth or on solidified agar plates containing 1.5% (w v^−1^) agar. Wild type and all Syn2973 mutants constructed were routinely grown in BG11 medium or agar plates containing 1.5% (w v^−1^) agar at 38 °C in shaking incubators at 130 rpm under an illumination intensity of approximately 400 μmol photons m^−2^ s^−1^. When necessary, media were supplemented with 10 μg ml^−1^ chloromycetin or kanamycin. The growth of Syn2973 (OD_730_) was measured using a UV-1000 spectrophotometer (PERSEE, China) at selected time intervals.

For nitrogen and phosphate addition assays, the mutant strain Syn2973-LdhOmcS was inoculated in 100 ml flasks containing 30 ml BG11 medium at an initial OD_730_ of 0.1, grown at 38 °C in shaking incubators at 130 rpm under an illumination intensity of approximately 400 μmol photons m^−2^ s^−1^. After 24 h cultivation, except for the control group Syn2973-LdhOmcS-1NP (containing 17.65 mM NaNO_3_ and 0.175 mM K_2_HPO_4_), other groups of cultures were supplemented with different concentration of NaNO_3_ and K_2_HPO_4_ to make nitrate-replete and phosphate-replete media. The group Syn2973-LdhOmcS-2NP contains 35.3 mM NaNO_3_ and 0.351 mM K_2_HPO_4_, the group Syn2973-LdhOmcS-4NP contains 70.6 mM NaNO_3_ and 0.701 mM K_2_HPO_4_, the group Syn2973-LdhOmcS-8NP contains 141.2 mM NaNO_3_ and 1.402 mM K_2_HPO_4_. Without extra addition of K_2_HPO_4_, the groups Syn2973-LdhOmcS-2 N, Syn2973-LdhOmcS-4NP and Syn2973-LdhOmcS-8NP contains 35.3 mM NaNO_3_, 70.6 mM NaNO_3_ and 141.2 mM NaNO_3_, respectively. The growth of the strain Syn2973-LdhOmcS in different BG11 media was monitored by measuring OD_730_.

### Construction of mutant strains

*E. coli* DH5α was used for vector construction and amplification. All plasmids used in mutant construction were list in Additional file 1: Table S6 and the related primers used in plasmids construction were listed in Additional file 1: Table S7. The plasmid pSyn2973-Ldh was constructed by inserting the P_cpc560_-*ldh*-T_rbcS_ expression cassette into two flanking homologous regions *nbla* up and *nbla* down which had been cloned into the HindIII and the BamHI restriction sites of the plasmid pBR322. The P_cpc560_-*ldh*-T_rbcS_ expression cassette consists of a strong Promoter P_cpc560_ [42], a structural gene *ldh* from *Lactobacillus delbrueckii* ATCC 11,842, and a terminator T_rbcS_. The plasmid pSyn2973-ΔNbla was constructed by inserting the *nbla* knockout cassette into the plasmid pBR322 under the HindIII and the BamHI restriction sites. The plasmid pSyn2973-ΔGlcD1 was constructed by inserting the *glcD1* knockout cassette into the plasmid pBR322 under the HindIII and the BamHI restriction sites. The plasmid pSyn2973-LdhOmcS was constructed by inserting the P_rbcL200_-*omcS*-T_rbcS_ expression cassette between two flanking homologous regions *glcD1* up and *glcD1* down which had been cloned into the HindIII and the BamHI restriction sites of the plasmid pBR322. The P_rbcL200_-*omcS*-T_rbcS_ expression cassette consists of a promoter P_rbcL200_, a structural gene *omcS* [27], and a terminator T_rbcS_ (Additional file 1: Table S6).

Mutants of Syn2973 were constructed by Tri-parental conjugation as previously described with minor modifications [26]. *E. coli* HB101 carrying both the target plasmid and the pRL623, *E. coli* HB101 carrying the pRL443 and wild type of Syn2973 were cultivated overnight. 100 μl of each *E. coli* cells and 200 μl of Syn2973 cells were washed twice with BG11 medium and then mixed together. Mixed cells were plated onto sterile filters resting on the BG11 + 5% LB (w v^−1^) + 50 mM NaHCO_3_ agar plates without antibiotics. After 12 h incubation at 38 °C under 200 μmol photons m^−2^ s^−1^ continuous illumination, the filters were transported onto the selective BG11 plates with 10 μg ml^−1^ chloromycetin and/or kanamycin. Mutant colonies were usually visible within 4 days. Correct recombinants were verified by PCR and sequencing.

### Reverse-transcription PCR

RT-PCR was performed as previously described [55]. Total RNA of 10 OD_730_ fresh cells was extracted with the RNAprep Pure Plant Kit (Tiangen Biotech, Beijing, China). Residual DNA in RNA was removed by the the RNase-free Dnase I (Tiangen Biotech, Beijing, China). Reverse transcription reactions using random primers and Oligo dT primers were performed with FastKing gDNA Dispelling RT SuperMix Kit (Tiangen Biotech, Beijing, China). Possible DNA contamination was verified without reverse transcriptase (RT) enzyme. Reverse transcription products were amplified by PCR and analyzed by electrophoresis on 1.2% (w v^−1^) agarose gels.

### Electrochemical assays

The photocurrent generation by the Syn2973, Syn2973-ΔNbla and Syn2973-OmcS were conducted using a CHI1030C potentiostat (CH Instruments, China) as previously described (Sekar et al., 2014) with modifications. Ag/AgCl/Sat. KCl was used as the reference electrode, a carbon cloth (3 × 3 cm) was used as the counter electrode, a carbon cloth (2.5 × 2.5 cm) was used as the anode (working electrode). 100 mM PBS (pH 7.0) buffer was used as the catholyte. BG11 was used as anolyte in the full cell. The photocurrent generation was measured by applying an over-potential of 0.3 V (vs. Ag/AgCl) for Syn2973, Syn2973-ΔNbla and Syn2973-OmcS.

For the inhibitor assays, the fresh cells were harvested by centrifugation (9,000 rpm, 8 min) and resuspended in fresh BG11 medium with an OD_730_ of 1.0. The cells were incubated with the inhibitor 0.5 mM 3-(3,4-Dichlorophenyl)-1,1-dimethylurea (DCMU) or 0.5 mM 2,5-dibromo-3-methyl-6-isopropylbenzoquinone (DBMIB) before the photocurrent was measured [37]. Solutions of inhibitors DCMU and DBMIB were prepared with 100% ethanol.

### Measurements of chlorophyll fluorescence of photosystems

The chlorophyll fluorescence parameters were measured using a Dual-PAM-100 fluorometer (Walz, Germany) as previously reported [56]. In brief, 2 ml cell suspension at an OD_730_ of 6.0 with or without 0.5 μM DBMIB were measured. Slow induced curve and light response curve were monitored to get the chlorophyll fluorescence kinetic parameters, including the relative electron transport rate (rETR(II) and rETR(I)) and the effective quantum yield (Y(II) and Y(I)).

### Quantification of NADH, NAD^+^, NADPH, NADP^+^, ATP and ADP

20 ml of wild-type and mutants Syn2973 grown to an OD_730_ of 1.0 were harvested by centrifugation (8,000 rpm, 3 min, 4 °C) and washed with cold PBS buffer twice. The cells were resuspended in 600 μl NAD^+^/NADH extraction buffer or in 600 μl NADP^+^/NADPH extraction buffer, respectively. Cells were disrupted by an ultrasonic cell disruptor for 10 min at 0 °C. The supernatant was used for NADH, NAD^+^ or NADPH, NADP^+^ determination.

The intracellular NADH, NAD^+^ and NADPH, NADP^+^ were determined by using NAD^+^/NADH Assay Kit with WST-8 (Beyotime Biotech, Shanghai, China) and NADP^+^/NADPH Assay Kit with WST-8 (Beyotime Biotech, Shanghai, China) according to the manufacturer’s instruction, respectively. The concentrations of NADH, NAD^+^ and NADPH, NADP^+^ were calculated according to the NADH and NADPH standard curve, respectively.

The intracellular ATP and ADP concentrations were determined by using the ADP/ATP Ratio Assay Kit (Sigma-Aldrich, USA)[57] according to the manufacturer’s instruction. The ATP and ADP concentrations were calculated according to the ATP standard curve.

### d-lactate quantification

For lactate fermentation assay, the wild-type and mutants Syn2973 were grown in 100 ml flasks containing 30 ml BG11 medium with an initial OD_730_ of 0.1 at 38 °C in a shaking incubators at 130 rpm under an illumination intensity of approximately 400 μmol photons m^−2^ s^−1^. Growth was monitored by measuring OD_730_.

For measurement of extracellular d-lactate concentration, 500 μl batch cultured samples were harvested at selective time-points. Cells were removed by centrifugation at 14,000 g for 5 min, the supernatant was filtered and then analyzed by HPLC (Agilent 1260 Infinity Series) similar to previously reported [39]. The d-lactate concentration was calculated according to the d-lactate standard curve.

### Extraction and determination of chlorophyll a

Cells from exponential growth phage were harvested by centrifugation (8,500 g, 10 min) and suspended in 1% Mg(HCO_3_)_2_ solution. The pellets were re-suspended in 90% acetone (v v^−1^) overnight to extract the chlorophyll a. The extraction mixtures were centrifuged at 8,500 g for 15 min and the supernatant was used to determine the concentration of chlorophyll a. The concentration of chlorophyll a (Chl a) was calculated by monitoring the OD_630_, OD_645_, OD_663,_ and OD_750_ according to the formula [58]:

$$ {\text{Chla }}(\mu {\text{g ml}}^{{ - {1}}} )\, = \,{11}.{64}*\left( {{\text{OD}}_{{{663}}} - {\text{OD}}_{{{75}0}} } \right) - {2}.{16}*\left( {{\text{OD}}_{{{645}}} - {\text{OD}}_{{{75}0}} } \right)\, + \,0.{1}*({\text{OD}}_{{{63}0}} - {\text{OD}}_{{{75}0}} ) $$

### Oxygen evolution

The photosynthetic oxygen evolution rate were determined using a Chlorolab2 Liquid-Phase Electrode (Hansatech) according to previous reports [59, 60]. Cells at exponential growth phase were harvested and re-suspended in 2 mL BG11 medium containing 100 mM bicarbonate at an OD_730_ of 6. The light was provided at various intensities following a cycle of 2 min of constant illumination and 5 min darkness after 5 min initial dark pretreatment. Light intensity included 0, 200, 500, 1000, 1500, 2000 μmol photons m^−2^ s^−1^. The O_2_ evolution rates were calculated according to the concentration of chlorophyll a (Additional file 1: Table S8).

### Transcriptome analysis

Syn2973, Syn2973-Ldh, and Syn2973-LdhOmcS grown to an OD_730_ of 1.5 were harvested by centrifugation (12,000 g, 5 min) and washed with fresh BG11 medium for one time. 0.2 OD_730_ of cells were suspended with fresh BG11 medium containing 50 mM NaHCO_3_ in 100 mL flasks and cultured at 38 °C in a shaking incubator at 130 rpm under an illumination intensity of approximately 400 μmol photons m^−2^ s^−1^. Growth was monitored by measuring OD_730_. 10 OD_730_ batch cultured samples were harvested by centrifugation (12,000 g, 10 min) at selective time-points (12 h and 36 h) at 4 °C and washed with pre-cooled PBS once, and immediately cooled down with liquid nitrogen for 30 s, and finally stored in -80 °C. The samples at different time point were sent to Igenecode Company (Beijing, China) for mRNA extraction and transcriptome analysis. mRNA was fragmented within fragmentation buffer, then used as templates for cDNA synthesis and cDNA library construction. The library was sequenced using Illumina HiSeq™ 2000 [61]. The raw reads were filtered into clean reads and aligned to the reference genes and genome files using SOAP2 (http://soap.genomics.org.cn/). Differential expression of genes between treatments and control groups were identified using DEseq2 (https://bioconductor.org/packages/release/bioc/vignettes/DESeq2/inst/doc/DESeq2.html) [62]. Gene Ontology (GO) enrichment analyses for each protein-coding gene of UTEX 2973 were obtained using Blast2GO v4.1.7 (https://www.blast2go.com/). Kyoto Encyclopedia of Genes and Genomes (KEGG) pathway enrichment was performed enrichment analyses were performed to identify significantly enriched metabolic pathways in differentially expressed genes (DEGs) with a magnitude of Log_2_Ratio ≥ 1 and false discovery rate ≤ 0.001 using clusterProfiler (https://bioconductor.org/packages/release/bioc/html/clusterProfiler.html) [62].

## Supplementary Information

**Additional file 1.** Additional tables.

## Data Availability

Data are available from the corresponding author on reasonable request.

## Abbreviations

PBS: Phycobilisome, composed of allophycocyanin (AP), phycocyanin (PC)/phycoerythrocyanine (PEC) and phycoerythrin (PE)
PSII: Photosystem II
PSI: Photosystem I
PQ: Plastoquinone
Cyt*b*_*6*_*f*: Cytochrome b_6_f complex
PC: Plastocyanin
NDH: NAD(P)H-quinone oxidoreductase complex
Fd: Ferredoxin
FNR: Ferredoxin-NADP^+^ reductase
CoxC: Cytochrome c oxidase complex
OmcS: Outer membrane cytochrome S
d-Ldh: d-lactate dehydrogenase. Calvin cycle
RBCL/S: Ribulose-bisphosphate carboxylase large/small subunits
Rpe: Ribulose-phosphate 3-epimerase
Prk: Phosphoribulokinase
TCA CYCLE: Tricarboxylic cycle
GltA: Citrate synthase
Ogdh: 2-Oxoglutarate dehydrogenase E1 component
Icd: Isocitrate dehydrogenase. OPP, Pentose phosphate pathway
Fbp: Fructose-1,6-bisphosphatase I
GlpX: Fructose-1,6-bisphosphatase II
Pgi: Glucose-6-phosphate isomerase
TktA/B: Transketolase
GAPDH: Glyceraldehyde 3-phosphate dehydrogenase
GapA: Glyceraldehyde-3-phosphate dehydrogenase (NADP^+^)
Pgk: Phosphoglycerate kinase
GpmA: 2,3-Bisphosphoglycerate-dependent phosphoglycerate mutase
