# Supplementary material for: Over-expression of an electron transport protein OmcS provides sufficient NADH for d-lactate production in cyanobacterium
Source: Biotechnol Biofuels. 2021 Apr 29;14:109. doi: 10.1186/s13068-021-01956-4 (PMC8082822; doi:10.1186/s13068-021-01956-4)
Supplement: Supplementary file 1 — Additional file 1. Additional tables. [file 13068_2021_1956_MOESM1_ESM.pdf]

# Over-expression of an electron transport protein OmcS provides sufficient NADH for D-lactate production in cyanobacterium

Hengkai Meng†[a,b], Wei Zhang†[a,b], Huawei Zhu[b,c], Fan Yang[b,c], Yanping Zhang[b], Jie Zhou[b]*, Yin Li[b]*

**Table S1** Transcript abundance of genes involved in photosynthesis antenna pathway

| Pathway or protein | Gene name | Gene ID | $Log_2$Ratio | | | |
|---|---|---|---|---|---|---|
| | | | Syn2973-Ldh-12h/Syn2973-12h | Syn2973-Ldh-36h/Syn2973-36h | Syn2973-LdhOmcS-12h/Syn2973-12h | Syn2973-LdhOmcS-36h/Syn2973-36h |
| Allophycocyanin (Ap) | | | | | | |
| | *apcA* | M744_RS12140 | 1.9 | | 1.3 | 1.7 |
| | *apcB* | M744_RS01320 | | 1.2 | | 1.8 |
| | *apcC* | M744_RS01325 | | 1.3 | | 1.7 |
| | *apcD* | M744_RS01760 | 1.1 | 1.3 | 1.4 | 1.9 |
| | *apcE* | M744_RS01310 | | 1.4 | | 1 |
| | *apcF* | M744_RS05335 | -1.3 | | 1.2 | 1.6 |
| Phycocyanin (PC)/Phycoerythrocyanine (PEC) | | | | | | |
| | *cpcC* | M744_RS10910 | -1.9 | -1.2 | | |
| | *cpcE* | M744_RS10885 | -3.4 | -1.2 | 1.4 | 2.2 |
| | *cpcF* | M744_RS10880 | -2.4 | | | 1.9 |

| | | | | | | |
|---|---|---|---|---|---|---|
| | *cpcG* | M744_RS05335 | | | 1.2 | |
| Phycoerythin (PE) | | | | | | |
| | *cpeS* | M744_RS05335 | -1.3 | -2.9 | 1.9 | -1.2 |
| | *cpeT* | M744_RS12150 | -1.9 | -2.9 | | 1 |
| | *cpeU* | M744_RS07380 | -1.1 | -1.7 | | |

(the Magnitude of $Log_2$Ratio ≥ 1; Map00196)

**Table S2** Transcript abundance of genes involved in photosynthesis

| Pathway or protein | Gene name | Gene ID | Log$_2$Ratio | | | |
|---|---|---|---|---|---|---|
| | | | Syn2973-Ldh-12h/Syn2973-12h | Syn2973-Ldh-36h/Syn2973-36h | Syn2973-LdhOmcS-12h/Syn2973-12h | Syn2973-LdhOmcS-36h/Syn2973-36h |
| PSII | | | | | | |
| | *psbA* | M744_RS10235 | -1.5 | | 1.2 | |
| | *psbD* | M744_RS07945 | | | -1.6 | |
| | *psbC* (cp43) | M744_RS09425 | -1.0 | | | -2.0 |
| | *psbE* (cp47) | M744_RS10260 | -1.5 | | | |
| | *psbO* | M744_RS01480 | -1.9 | | 1.2 | 2.3 |
| | *psbV* | M744_RS06100 | | | 1.4 | 1.8 |
| | *psb27* | M744_RS01230 | | -1.1 | 1.4 | |
| | *psb28* | M744_RS07740 | 1.3 | | 1.2 | |
| | *psb28-2* | M744_RS03700 | | | | -1.3 |
| PSI | | | | | | |
| | *psaC* | M744_RS00265 | | | 1.7 | 1.6 |
| | *psaD* | M744_RS11145 | -1.9 | | | |
| | *psaF* | M744_RS09885 | -2.2 | | 1.1 | 1.1 |
| | *psaL* | M744_RS04405 | -1.6 | | | |
| | *psaE* | M744_RS09530 | | | | -1.1 |
| Cytochrome b6/f complex | | | | | | |
| | *petA* | M744_RS09980 | | | 1.9 | 1.6 |
| | *petC* | M744_RS09975 | -1.7 | -1.5 | 1.9 | 1.8 |
| Photosynthetic electron transport | | | | | | |

| | | | | | |
|---|---|---|---|---|---|
| *petH* (FNR) | M744_RS11270 | | -1 | | |
| *petE* (PC) | M744_RS10705 | | | 1.4 | |
| *petJ* (cyt c6) | M744_RS08785 | | | 1.9 | 2.4 |
| *petF* (Fd) | M744_RS08130 | | | | -2 |

(the Magnitude of $Log_2$Ratio ≥ 1; Map00195)

**Table S3** Transcript abundance of genes involved in oxidative phosphorylation

| Pathway or protein | Gene name | Gene ID | Log$_2$Ratio | | | |
|---|---|---|---|---|---|---|
| | | | Syn2973-Ldh-12h/Syn2973-12h | Syn2973-Ldh-36h/Syn2973-36h | Syn2973-LdhOmcS-12h/Syn2973-12h | Syn2973-LdhOmcS-36h/Syn2973-36h |
| NADH dehydrogenases | | | | | | |
| | *ndhJ* | M744_RS10235 | | -1.1 | | |
| | *ndhL* | M744_RS00870 | | -1.2 | | -1.3 |
| | *ndhA* | M744_RS09425 | | -1.2 | | |
| | *ndhI* | M744_RS09420 | | -1.6 | | |
| | *ndhE* | M744_RS09410 | | -1.7 | | |
| | *ndhD* | M744_RS08940 | | -1.2 | | |
| | *ndhN* | M744_RS04960 | | | | 1.4 |
| | *ndhG* | M744_RS09415 | -1.3 | -1.8 | | |
| | *ndhF* | M744_RS06265 | | 1.2 | 2.0 | 2.3 |
| | *nuoF* | M744_RS05500 | | -1.0 | | |
| Cytochrome C oxidase | | | | | | |
| | *cybd* | M744_RS07320 | | -1.4 | | |
| | *coxC/ctaE* | M744_RS03025 | | | -2.0 | |
| ATP synthesis | | | | | | |
| | *ppk* | M744_RS08155 | 1.2 | -1.1 | 1.7 | |
| | *ppa* | M744_RS09220 | | -1.5 | 1.1 | 1.4 |
| F-type ATPase, F1 | | | | | | |
| | *atpA* | M744_RS01270 | -1.4 | -2.0 | 1.0 | 1.5 |

| | | | | | | |
|---|---|---|---|---|---|---|
| | *atpD* | M744_RS04535 | -1.5 | -1.8 | 1.0 | 1.4 |
| | *atpH* | M744_RS01275 | -1.7 | -2.8 | 1.7 | 1.2 |
| | *atpG* | M744_RS01265 | | | | 1.6 |
| | *atpC* | M744_RS04530 | -1.2 | -2.0 | 1.5 | |
| F-type ATPase, F0 | | | | | | |
| | *atpF* | M744_RS01280 | -1.1 | -1.8 | | 1.4 |
| | *atpE* | M744_RS01290 | | | | 1.2 |

(the Magnitude of $Log_2Ratio \geq 1$; Map00190)

**Table S4** Transcript abundance of genes involved in carbon metabolism

| Pathway or protein | Gene name | Gene ID | Log$_2$Ratio | | | |
|---|---|---|---|---|---|---|
| | | | Syn2973-Ldh-12h/Syn2973-12h | Syn2973-Ldh-36h/Syn2973-36h | Syn2973-LdhOmcS 12h/Syn2973-12h | Syn2973-LdhOmcS-36h/Syn2973-36h |
| Glycolysis | | | | | | |
| | *gcvPA* | M744_RS10155 | -1.3 | -1.4 | | |
| | *gapDH* | M744_RS06450 | | -5.4 | | |
| | *gapA* | M744_RS07430 | | -2.1 | 1.5 | |
| | *pgk* | M744_RS10570 | | -1.3 | 1.3 | |
| | *gpmA* | M744_RS00590 | | -1.8 | | |
| Pyruvate metabolism | | | | | | |
| | *ppc* | M744_RS04855 | | -1.2 | | |
| | *pps/ppsA* | M744_RS12240 | | 2.2 | -1.1 | |
| | *porA* | M744_RS04190 | | 1.7 | -1.1 | |
| | *aceE* | M744_RS11680 | -3.4 | -2 | | |
| | | M744_RS10155 | -1.3 | | | |
| | *aceE* | M744_RS10810 | | | | 1.4 |
| | *aceE* | M744_RS02255 | | | 1.1 | |
| | *ackA* | M744_RS05755 | -1.6 | -2 | | -1.3 |
| | *acs* | M744_RS09380 | 1.4 | 1.9 | | 1.2 |
| | *accB* | M744_RS09240 | 1.1 | -1.6 | 1.1 | 1.8 |
| | *accC* | M744_RS06365 | 1 | | | |
| TCA cycle | | | | | | |

| | | | | | | |
|---|---|---|---|---|---|---|
| | *cs/gltA* | M744_RS13130 | 2 | | 1.7 | |
| | *ogdh/sucA* | M744_RS11680 | -3.4 | -1.4 | | |
| | | M744_RS10155 | -1.3 | | | |
| | *icd* | M744_RS07555 | | | 1 | |
| Calvin cycle | | | | | | |
| Rubisco | *rbcL* | M744_RS09005 | -1.8 | -1.3 | | |
| | *rbcS* | M744_RS09000 | -1.2 | -1.3 | | |
| | *rpe* | M744_RS13170 | 1 | | 1.2 | 1.1 |
| | *prk* | M744_RS11275 | | -1.5 | 1.4 | |
| Pentose phosphate pathway | | | | | | |
| | *fbp* | M744_RS04435 | 1.2 | -2.3 | | |
| | *glpX* | M744_RS00415 | -1.1 | -2.5 | | 1.1 |
| | *pgi* | M744_RS06000 | | | | 1.3 |
| | *tktA, tktB* | M744_RS00250 | -2.3 | -2.5 | 1.2 | 1.1 |

(the Magnitude of $Log_2$Ratio ≥ 1; Map0120)

**Table S5** Strains used and constructed in this study

| Strains | Genotype | Origin |
|---|---|---|
| *E. coli* | | |
| *E. coli* DH5a | Cloning host strain | Transgene Co., Ltd |
| *E. coli* HB101 (pRL623) | Conjugation helper strain | This study |
| *E. coli* HB101 (pRL443) | Conjugation strain | This study |
| Cyanobacteria | | |
| WT | Wild-type *Synechococcus elongatus* UTEX 2973 | Himadri B. Pakrasi |
| Syn2973-ΔNbla | *nbla*::*Cm*$^{r}$ | This study |
| Syn2973-OmcS | *nbla*::$P_{rbcL200}$-*omcS*, *Cm*$^{r}$ | This study |
| Syn2973-Ldh | *nbla*::$P_{cpc560}$-*ldh*, *Cm*$^{r}$ | This study |
| Syn2973-LdhΔGlcD1 | *nbla*::$P_{cpc560}$-*ldh*, *Cm*$^{r}$; *glcD1*::*Kan*$^{r}$ | This study |
| Syn2973-LdhOmcS | *nbla*::$P_{cpc560}$-*ldh*, *Cm*$^{r}$; *glcD1*::$P_{rbcL200}$-*omcS*, *Kan*$^{r}$ | This study |

**Table S6** Plasmids used and constructed in this study

| Plasmids | Description | Origin |
|---|---|---|
| pSyn2973-ΔNbla | pBR322 derivated, containing *nbla* knockout cassette, *Amp*$^r$ *Cm*$^r$. | This study |
| pSyn2973-OmcS | Integration vector at *nbla* locus with $P_{rbcl200}$-*omcS* expression cassette, *Amp*$^r$ *Cm*$^r$. | This study |
| pSyn2973-Ldh | Integration vector at *nbla* locus with $P_{cpc560}$-*ldh* expression cassette, *Amp*$^r$ *Cm*$^r$. | This study |
| pSyn2973-ΔGlcD1 | pBR322 derivated, containing *glcD1* knockout cassette, *Amp*$^r$ *Kan*$^r$. | This study |
| pSyn2973-LdhOmcS | Integration vector at *glcD1* locus with $P_{rbcl200}$-*omcS* expression cassette, *Amp*$^r$ *Kan*$^r$. | This study |

**Table S7** Primers used in this study

| Names | Sequence |
|---|---|
| *nbla up*-F | cgatAAGCTTgatgcgcagatagcctgact |
| *nbla up*-R | tggACTAGTgggagcctccggcactgcag |
| *Cm*-F | gttgataatgaactgtgctg |
| *Cm*-R | atcgaatttctgccattcat |
| *nbla down*-F | accgtgtgcaagacttgccc |
| *nbla down*-R | tagaGGATCCatgctgctggagttctacgc |
| $P_{cpc560}$-F | tcccACTAGTacctgtagagaagagtccct |
| $P_{cpc560}$-R | tgaattaatctcctacttga |
| *RbcL*-F | atgcccaagacgcaatctgc |
| *RbcL*-R | ttagagcttgtccatcgtt |
| *ldh*-F | atgactaaaatcttcgcct |
| *ldh*-R | ttaacccactttaaccggcg |
| $T_{rbcS}$ of *ldh*-F | gttacagttttggcaattac |
| $T_{rbcS}$ of *ldh*-R | caacGGTACCtaattgacaattgacaattc |
| *glcD1 up*-F | GAATTCttctatctaagcatcaagtg |
| *glcD1 up*-R | ttcttgacgagttcttctgagagctggaatttcagtcacg |
| *Kan*-F | cgtgactgaaattccagctctcagaagaactcgtcaagaa |
| *Kan*-R | gtctGGTACCcgatGATATCcaagcgaaccggaattgcca |
| *glcD1 down*-F | cttgGATATCatcgGGTACCagacgaactgttgcctatgc |
| *glcD1 down*-R | atcgGTCGACcgggatgccctcgacatcga |
| $P_{rbcL200}$-F | gtctGGTACCgggcttcaataaatggttcc |
| $P_{rbcL200}$-R | atgcccttcttcatggccatgtcgtctctccctagagata |
| *omcS*-F | tatctctagggagagacgacatggccatgaagaagggcat |

| | |
|---|---|
| *omcS*-R | gtaattgccaaaactgtaacctaatccttggcgtgacact |
| $T_{rbcS}$ of *omcS*-F | agtgtcacgccaaggattaggttacagttttggcaattac |
| $T_{rbcS}$ of *omcS*-R | cttgGATATCtaattgacaattgacaattc |

**Table S8** Intracellular contents of Chl a in strains

| Strain | Chl a (μg $OD_{730}^{-1}$) |
|---|---|
| WT | 6.1±0.1 |
| Syn2973-OmcS | 5.9±0.1 |
| Syn2973-Ldh | 5.7±0.2 |
| Syn2973-LdhΔGlcD1 | 5.7±0.3 |
| Syn2973-LdhOmcS | 5.9±0.2 |

Data shown are mean of three independent measurements.
